# The effects of adapted physical education sessions on the empathy of female students with overweight

**DOI:** 10.3389/fpsyg.2023.1170446

**Published:** 2023-05-31

**Authors:** Oumayma Slimi, Santo Marsigliante, Vito Ciardo, Mourad Bahloul, Okba Selmi, Nidhal Jebabli, Antonella Muscella

**Affiliations:** ^1^Higher Institute of Sport and Physical Education of Sfax, University of Sfax, Sfax, Tunisia; ^2^Department of Biological and Environmental Sciences and Technologies (Di.S.Te.B.A.), University of Salento, Lecce, Italy; ^3^Department of Biological and Environmental Science and Technologies, University of Salento, Lecce, Italy; ^4^Higher Institute of Education and Continuing Training, Virtual University of Tunis, Tunis, Tunisia; ^5^Higher Institute of Business Administration of Sfax, University of Sfax, Sfax, Tunisia; ^6^Higher Institute of Sports and Physical Education of Kef, University of Jendouba, Kef, Tunisia

**Keywords:** obesity, physical education, adaptation, empathy, emotional splitting

## Abstract

The global prevalence of childhood and adolescent overweight and obesity increases rapidly. Physical activity plays a major role in the prevention of obesity. The present study aimed to analyze the effect of adapted basketball sessions according to the empathic capacity of adolescent girls with overweight. Forty-two girls with overweight (age: 16.09 ± 0.85; years; height: 1.64 ± 0.67 m: weight: 73.02 ± 0.61 kg; BMI: 27.15 ± 1.37) volunteered to participate in the study and were randomly assigned to the experimental group (EG, *n* = 21) and control group (CG, *n* = 21). EG was submitted to a basketball intervention adapted to students with obesity while the CG performed classic basketball exercises for 7 weeks. Each week girls had 2 basketball teaching-learning sessions, lasting 50 min. The participants’ empathy was assessed before and after the intervention using the Favre CEC. The results showed that adaptation intervention was associated with a significant emotional contagion decrease (Δ% = 0.466) and splitting with emotions (Δ% = 0.375), and with an empathy increase (Δ% = 1.387), in EG compared to CG. No significant difference was assessed in the empathy CG, before and after the intervention. This study demonstrated that adapted physical education classes could be an effective strategy to improve empathetic skills and inclusion of overweight girls as well as a means to prevent obesity.

## 1. Introduction

The increasing prevalence of obesity and overweight in the pediatric population is a serious public health problem, causing multiple and harmful consequences (Shawon et al., 2020; Jha and Mehendale, 2022). According to the World Health Organization (WHO), when body mass index (BMI) is more than 25, the situation is denoted as overweight, and a BMI of more than 30 is considered an obesity condition (WHO, 2022). The conditions of overweight and obesity primarily occur due to energy imbalances between calories consumed, calories exhausted, and excessive calorie intake or insufficient physical activity. Childhood with overweight/obesity is a precursor to metabolic syndrome, poor physical health, mental disorders, respiratory problems, and glucose intolerance, which can continue into adulthood (Drozdz et al., 2021; Jankowska et al., 2021).

On average, 60% of children suffering from overweight/obesity have at least one additional risk factor for cardiovascular diseases, such as hypertension, hyperlipidemia, or hyperinsulinemia (Močnik and Marčun, 2021). The risk factor for developing abnormal lipid profiles is high among children with overweight/obesity (Ruano et al., 2018; McPhee et al., 2020).

The higher prevalence of childhood and adolescent with overweight and obesity is mainly due to the reduced level of physical activity in the contemporary pediatric population (Tremblay et al., 2011; Tabacchi et al., 2016; Di Maglie et al., 2022; Marsigliante et al., 2022).

Physical activity, in particular physical exercise, remains the main intervention that can counteract obesity, being an important element in the prevention of excessive body mass (Strong et al., 2005; Guinhouya et al., 2013; Wyszyńska et al., 2020; Di Maglie et al., 2022; Marsigliante et al., 2023).

Lifestyle behaviors are established early in life, in childhood and adolescence, and once established, they persist into adulthood (Singh et al., 2008; Lioret et al., 2020). Thus, interventions to influence healthier lifestyles may be most effective when implemented in childhood, before unhealthy choices become ingrained in an individual’s lifestyle (van Sluijs et al., 2007; Subramaniam et al., 2022).

Since children and adolescents spend most of their day in schools, they provide the appropriate environment for implementing interventions that emphasize active and healthy lifestyles (Ip et al., 2017). One of the main objectives of physical education is the learning of correct and healthy lifestyles, an aim that becomes a necessity above all for students with obesity (Lirola et al., 2021).

However, as young people with overweight or obesity often have lower motor skills and physical condition than students with normal weight (Fogelholm et al., 2008; Li et al., 2013), they find many difficulties during physical education (PE) sessions. This leads them to perceive tiredness, tension, low self-esteem, incompetence, poor body image, and avoidance of exercise (Lynagh et al., 2015; Ievers-Landis et al., 2019). Hence, overweight, or obese students need a supportive environment around them, especially among their friends and teachers, free from stigma or negative attitudes (Peters and Ruan, 2010).

Since, as many studies have reported, children and adolescents with overweight and obesity experience more peer relationship problems than those with normal weight (Hestetun et al., 2015; Sönmez et al., 2019), in a physical education class, these students would face problems with many psychological barriers compared to children with normal weight (Thompson et al., 2007). Indeed, children and adolescents with obesity tend to show feelings of low self-esteem and self-limitation, social isolation, and stigmatization; sometimes they also develop a feeling that the physical education class is a hostile environment, thus creating negativity concerning their self-esteem and body image (Zabinski et al., 2003; Jodkowska et al., 2017).

However, students with obesity should be able to acquire skills and act like others; thus, teachers must facilitate a supportive environment for these overweight children, through effective strategies for integration and encouragement (Li et al., 2017; Aydi et al., 2023).

Given that the emotional competencies (e.g., empathy, emotional contagion, and splitting with emotions) are generally related to students learning (Jennings and Greenberg, 2009), it would be desirable to improve these emotional skills also, through physical activity.

Empathy is the ability to understand another person’s mood and emotional situation, without recourse to verbal communication (Davis, 1983). Whereby, empathy is a social skill of fundamental importance and represents one of the basic aptitudes for developing emotional competence, especially for fostering social ties, and effective interpersonal communication (Sánchez-Pérez et al., 2014). Empathy is the basis of successful interpersonal relationships, and it is the ability to establish stimulating and inspiring relationships; in addition, the empathic person contributes to their welfare. The concept of empathy has developed through the past century; different studies pointed out different concepts of empathy and its definition (Bošnjaković and Radionov, 2018). Empathy includes both affective and cognitive components (Davis, 1983). Emotional empathy concerns the experience of the emotions of others; while cognitive empathy concerns the ability to understand the perspective of others concerning emotional situations (Davis, 1983). Both components are essential to respond adequately to the emotional-expressive behavior of others. In addition to the cognitive and affective components, according to Hoffman, a third factor intervenes in the empathic experience: the motivational component (Hoffman, 2007). Therefore, the development of empathy, given the complexity of the cognitive mechanisms involved, has a gradual evolution, which is fully completed around the age of 13 (Hoffman, 2007).

Emotional empathy appears closely related to the functioning of mirror neurons. This system of neurons, identified for the first time in the premotor cortex of the macaque (area F5) by Italian researchers, (Gallese et al., 1996), has the particularity of being activated not only when the animal performs a given action, but also when it observes a similar one performing the same action.

Mirror neurons seem to be fundamental not only for the understanding of active processes and learning through imitation but also for the mechanisms of empathetic identification between similar individuals (Rizzolatti and Sinigaglia, 2010).

By the “perception-action of empathy” model of Preston and de Waal (2002), the perception of an emotional state in the other automatically activates in the observer the areas of representation of the emotion in question, generating an adequate emotional behavior, i.e., the ability to internally replicate aspects perceptual, motor, and emotional of the experiences lived by the observed person (Preston and de Waal, 2002).

Sports contexts are environments where people have numerous opportunities to engage in behaviors that have positive consequences for others; for example, during a team sport, it is possible to perceive the emotional signals of teammates, opponents, and coaches (Shima et al., 2021). At the same time, empathy could reduce aggression in sports by helping to maintain emotional and cognitive resonance with opponents and with one’s team during the competition (Baumeister and Lobbestael, 2011).

Unfortunately, while it is easy to find information on the causes of obesity, it is much more complicated to find adequate methods that can help teachers to act effectively with the student with obesity. Particularly, no previous studies have investigated the improvement of the empathic capacity of adolescents with obesity.

Therefore, this study attempts to fill this knowledge gap.

Since women with obesity disproportionately face poorer mental well-being, weight discrimination, and demotivated behaviors (Conradson et al., 2022), we directed our intervention to overweight girls to reduce social distance.

We have adapted the basketball exercises to promote socialization, interpersonal interactions, and communication as well as having fun; all of which would allow girls to develop emotional competence (Hoffman, 2007). This ability allows monitoring own feelings and those of others to achieve individual or common goals.

Thus, the purpose of this investigation aimed to analyze the effect of adapting PE sessions on the empathic capacity of overweight adolescent girls during a cycle of adapted basketball.

## 2. Materials and methods

### 2.1. Participants

To evaluate the effects of an intervention with adaptation (adapted basketball), using a direct comparison with a group that received an intervention without adaptation (classic basketball), we contacted a sample of regular different classes in a public secondary school, from the network of our research group through personnel contact.

We included schools that were willing to participate with a minimum of two classes.

Students and their parents received an informative letter about the study, including an informed consent form. We received the informed consent of 42 girls, who were included in the study. Written authorizations were also obtained from the heads of the schools.

The study was conducted according to the Declaration of Helsinki for human experimentation and approved by a local research ethics committee of the Higher Institute of Sport and physical education of Sfax (047/2022).

All the girls were healthy and free of any disabilities, musculoskeletal, cardiological, neurological, or respiratory diseases or dysfunctions, or any other conditions limiting their ability to perform the exercise and able to abstain from all physical activity outside the parameters of the study protocol during the test days. More specifically, the participants did not undertake new sports competitions or modifications to physical activities already practiced before this study, and for those who already practiced sports outside of school, the collaboration of their respective coaches was requested in order not to vary the workload.

The students were overweight or obese, following the thresholds proposed by the World Health Organization (World Health Organization, 1995) with a body mass index (BMI) of over 25 kg/m^2^ overweight and a BMI of more than 30 kg/m^2^ obese. The students were not suffering from any medical restrictions to participate in PE classes.

The girls, participating in the study, attended classes including both obese and overweight girls and boys, but also those with normal weight, with the same teacher, according to the same timetable so that no external factors interfered with the results.

### 2.2. Randomization

The randomized controlled trial study design was used; for its execution, we employed a “blind” statistical data analyst.

The 42 girls (15–17 years old) were randomly assigned to either the control group (CG, *n* = 21) or the experimental group (EG, *n* = 21).

A randomization procedure (a computer-generated list of random numbers using SPSS, version 24) was carried out by the lead researcher (OS, co-author). The basic information required for randomization (initials and participant code, age, weight, and height) was stored in a database.

Following the random assignment of the participants to the two groups (intervention and control), no significant differences emerged between these groups regarding the mean values of weight, height, and BMI (*p* > 0.05, according to Student’s *t*-test; Table 1).

**Table 1:** Anthropometric measurements.

Parameters	Mean (±SD)			
	EG (*n* = 21)	CG (*n* = 20)	Global (*n* = 41)	*p*-value
Age (years)	16.60 ± 0.76	16.12 ± 0.95	16.09 ± 0.854	0.78
Height (m)	1.63 ± 0.58	1.65 ± 0.75	1.64 ± 0.67	0.48
Weight (kg)	72.03 ± 0.45	74.02 ± 0.79	73.02 ± 0.61	0.13
BMI (kg.m^−2^)	27.11 ± 1.37	27.19 ± 1.4	27.15 ± 1.37	0.69

Data were expressed as means ± standard deviations. BMI, body mass index; EG, experimental group; CG, control group.

### 2.3. Experimental procedure

The study was to examine the effect of a 7 week physical activity intervention on overweight and obese students. Each week the EG students had 2 adapted basketball teaching-learning sessions, lasting 50 min.

**Figure 1:**
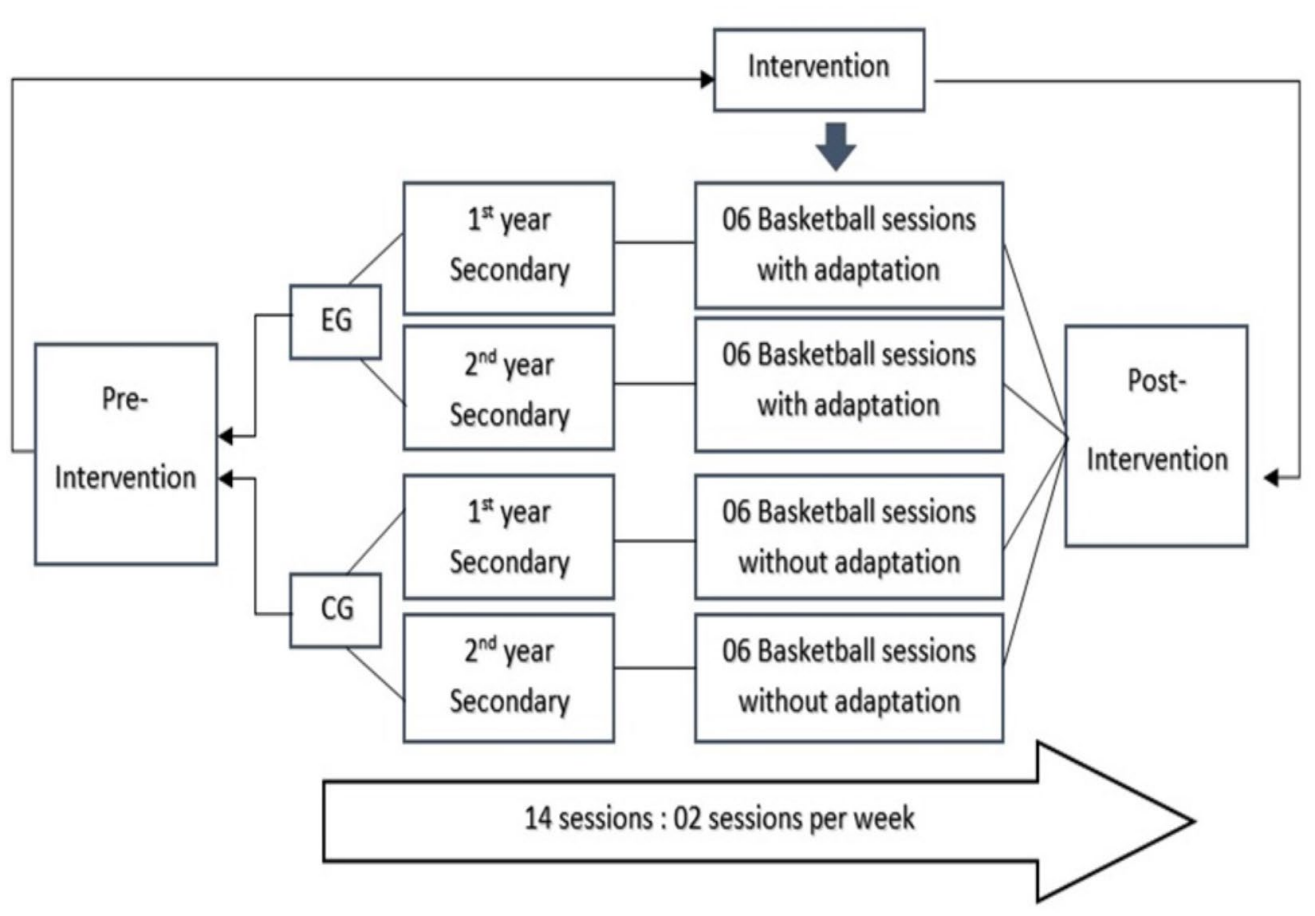
Experimental protocol design.

The intervention program was inspired by a pedagogical document previously designed by the Academic Group of Versailles in 2004 entitled “The pupil with obesity in EPS: an example of partial aptitude” from the “Adapted EPS and EPS and Handicap.” This document focused on adapted teaching contents in PE (Figure 1). The authors of the proposed document recommended some typical PE situations and how to adapt them to the student with obesity. Therefore, some of the exercises described previously in the document were used to construct an adapted basketball intervention. The methodological tool in this research was didactic engineering, proposing and then negotiating a didactic script (Carnus, 2009), based on adapted situations with the teachers associated with the research and monitoring their empathy with the students with obesity or overweight. Before the start of each experiment, the participants were familiarized with the material and the experimental protocol to reduce learning effects and to ensure that no external factors biased the results; the sessions were carried out by the same teachers, at the same time of the day and in the same spaces.

During the 7 weeks of intervention, students in the control group, performed 2 canonic basketball teaching-learning sessions per week, lasting 50 min, each.

### 2.4. Adapted intervention

During the intervention, the students normally followed the lessons with their teachers. In the first sessions, short and intense work phases alternated with active recovery phases (Brandou et al., 2003; Lazzer et al., 2010; Sween et al., 2014). During these sessions, the adolescents could enjoy playing regardless of their fitness level, as the competitive nature of the games played was minimized.

Subsequently, the exercises were adapted to make the game easier for obese students with overweight or obesity.

In 1vs1 basketball drills, to favor the game of pupils with obesity/overweight when they were in attack, the defender kept one hand behind his back and could start the game after the attacker had made the pass. 3v3 drills have also been changed to a 3v2 game that favors attacks.

Additionally, students could interchange whenever their team was in attack, thus, students with obesity could recover more frequently.

### 2.5. Measures

#### 2.5.1. Anthropometric measurements

The measurements of height and body mass were made using a height meter and an electronic scale (Tanita, Tokyo, Japan). The body mass index (BMI) was calculated for each subject according to the following formula: BMI = body mass (kg)/Height^2^ (m).

#### 2.5.2. Physical fitness

To get information regarding the PA improvements, the participants completed, before and after the intervention, tests that have been frequently used in similar studies. Each test assesses different components of physical fitness (Stodden et al., 2015; Tabacchi et al., 2016; Di Maglie et al., 2022; Yip et al., 2022): the standing long jump test (Davies, 1990), Push-ups (Yip et al., 2022); Sit-ups (Zheng et al., 2023), and Vameval test (Cazorla and Léger, 1993).

##### 2.5.2.1. Standing long jump

The standing long jump also known as the standing broad jump test was used to assess lower limb explosive power (Davies, 1990). Participants were instructed to “jump as far forward as possible, ensuring a two-footed landing” by placing both feet behind a designated starting line. Distance (cm) was measured from the starting line to the rearmost surface of the foot upon landing. Three trials were completed, with the best trial used for data analysis.

##### 2.5.2.2. Push-ups

Students were asked to maintain a prone position, placing their palms and toes on the ground, and keeping their backs straight. They moved their bodies up and down by bending and stretching their arms.

##### 2.5.2.3. Sit-ups

Students were instructed to lie supine with their knees bent at right angles and their feet placed on the mat. The students’ arms were crossed across their chests, with their hands on opposite shoulders. Each student flexed their torso, lifted their back off the mat, and completed as many repetitions as possible. The number of repetitions was counted by the examiner.

##### 2.5.2.4. Vameval test

The Vameval is a cardiorespiratory fitness test, performed to obtain the maximal aerobic speed (MAV; Cazorla and Léger, 1993). The test was performed as previously described (Ouertatani et al., 2022).

### 2.6. Empathy test

The empathy test (Contagion, Empathy, splitting with emotions), is a questionnaire previously validated by Filisetti et al. (2006), allowing to distinguish between empathy and emotional contagion but also to contrast them with “splitting with emotions” (Filisetti et al., 2006). It includes 12 situations that adolescents commonly encounter. Each adolescent was asked, after a round of basketball, to tick one of the three proposed answers according to his or her way of reacting. Each choice of answers is in the register of emotional contagion or in that of empathy or the splitting of emotions. In this test, three scores are calculated for each subject, each ranging from 0 to 12: the number of responses in the register of emotional contagion, which is defined as the involuntary reproduction of the emotion of another person or persons almost identically (nature, intensity); the number of responses in the register of empathy, which is considered as the intentional reproduction of the representations of others associated with a partial reproduction of their emotions and the number of responses in the register of splitting with emotions constituting an almost total blockage of the reproduction processes involved in emotional contagion and empathy.

### 2.7. Statistical analysis

The data are presented in the text and tables as means and standard deviations and, in the figures, as means and standard errors. The statistical analysis was performed using SPSS statistical software (version 22, IBM corps., Armonk, NY, United States). The normality of data sets was checked and confirmed using the Shapiro–Wilks test.

The statistical analysis of the data was performed using mean ± SD. The comparison, in each empathy subscale, between the two groups (control group vs. adaptation group) was performed using Student’s *t*-test for independent samples in the case where the normality of the distributions was confirmed and by the non-parametric Mann–Whitney test in the other case. The comparison between the two periods (before and after the basketball intervention) was carried out using the Student’s *t*-test for matched samples or by the Wilcoxon test for the subscales (emotional contagion, empathy, and emotional disconnection). The significance level was set to *p* < 0.05.

## 3. Results

### 3.1. Physical fitness

Consistently with a random assignment of students to experimental and control groups, no significant differences between groups were found in mean values for weight, height, and BMI (*p* > 0.05 by Mann–Whitney test; Table 1).

After 7 weeks of intervention, the distances covered during the standing long jump test were similar between GC and EG (Table 2, *p* > 0.05). Furthermore, the number of sit-ups and push-ups performed by the EG in 1 min was the same as that of the GC (Table 2, *p* > 0.05). Finally, maximal aerobic velocity values were also similar for EG and GC (*p* > 0.05).

**Table 2:** Post-intervention physical variations.

Parameters	Mean (±SD)		
	Experimental group	Control group	*p*-value
Standing Long Jump (cm)	149.21 ± 0.79	148.74 ± 0.68	0.403
Sit-ups (n)	13.4 ± 9.91	12.65 ± 9.19	0.437
Push-ups (n)	10.81 ± 9.05	10.59 ± 8.33	0.469
MAV (km/h)	9.228 ± 1.673	9.34 ± 1.412	0.156

MAV, maximal aerobic velocity.

In conclusion, regarding physical fitness, no significant differences were identified between the EG and CG before and after intervention (*p* > 0.05, Table 2).

### 3.2. Girl’s emotional contagion, empathy, and emotion splitting

**Figure 2:**
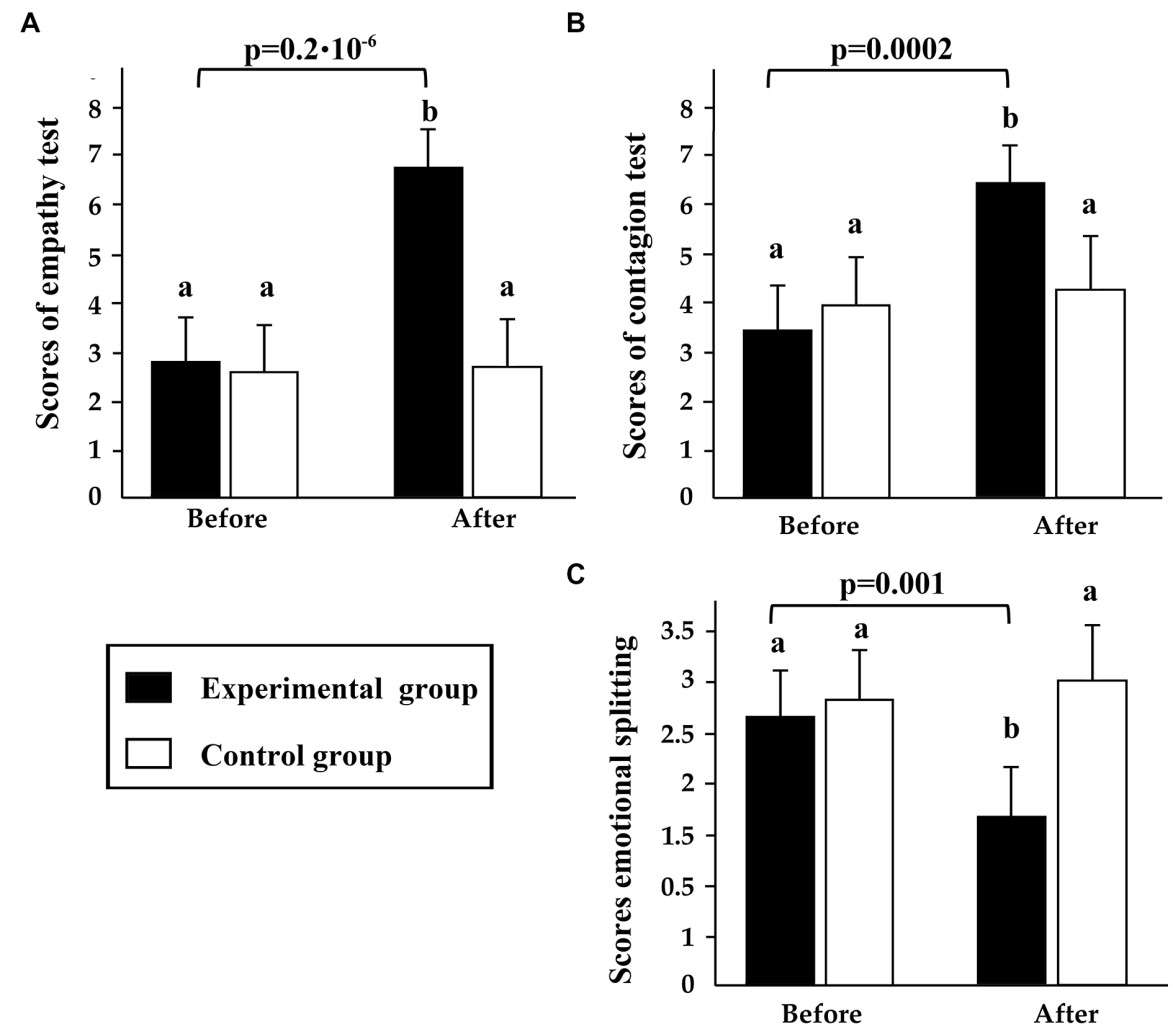
Variation in emotional contagion **(A)**, empathy **(B)**, and emotional splitting **(C)**, before and after the basketball intervention, in experimental (EG) and control group (CG) girls. Statistical analysis was carried out using the ANOVA: **(A)** and **(B)** *p* < 0.001, **(C)** *p* < 0.01. Coloums labeled with “a” lowercase letter (“CG” Before vs. “CG” After an “EG” Before vs. “CG” Before in **(A)**–**(C)**) are not significantly different according to Bonferroni/Dunn *post hoc* tests.

The level of emotional contagion was significantly higher after the adapted basketball intervention (Δ% = 0.466) in the EG, while no significant difference was recorded in the CG in the level of emotional contagion between before and after the intervention (Δ% = 0.045) (Figure 2A).

Regarding the difference between the groups in the same periods, the independent samples student’s *t*-test showed that the means of the CEC subscale scores were significantly higher for the CG compared to the EG (*p* < 0.05) before the intervention (Figure 2B).

The empathy scores in the EG were significantly higher after the adapted basketball intervention, compared to before the intervention (Δ% = 1.387); no significant difference was identified in the CG between before and after the intervention (Δ% = 0.045; Figure 2B).

In the EG, the level of emotional splitting was significantly lower after the intervention (Δ% = 0.375, Figure 2C); while no significant difference was identified in the CG in the level of emotional cut-off between before and after the intervention (Δ% = 0.063, Figure 2C).

After the intervention, emotion splitting was significantly higher for the CG compared to the EG (*p* < 0.05, Figure 2C).

## 4. Discussion

The main result of the present study is that the adapted basketball intervention was associated with an increase in emotional contagion and empathy and a decrease in splitting with emotions, in adolescent girls with obesity and overweight.

The increasing prevalence of obesity in the pediatric population is mainly due to the reduced level of physical activity in the contemporary young population (Močnik and Marčun, 2021). Thus, among the interventions, physical activity remains the main that can counteract obesity, being an important element in the prevention of excessive body mass in children and adolescents (Wyszyńska et al., 2020). In this context, and in the belief that PA interventions are useful for preventing or treating obesity and overweight, we examined the effect of a 7-weeks pilot intervention on girls with overweight from two secondary schools.

Since basketball training can improve the body composition of obese people, including young people and adolescents, was already known (Regaieg et al., 2013; Rinaldo et al., 2020; Liu and Kan, 2021) in our intervention, we suggested basketball training activities. Furthermore, basketball training, in addition to being positively associated with the physical performance and motor skills of players, in particular speed, agility, and explosive strength of the upper limbs (Rinaldo et al., 2020), can reduce the negative emotional state and effectively improve the mood of obese adolescents (Hagenaars et al., 2017). Precisely on this potential of basketball, the improvement of physical fitness was not one of the main objectives of our intervention, but it aimed to analyze the effect of adapted physical education sessions on the social and emotional skills of girls with obesity or overweight.

Adolescents with overweight or obesity experience a variety of discomforts when participating in physical activity (Stankov et al., 2012); some obstacles are potentially common to all adolescents; while others, such as physical discomfort and fatigue, are related to their weight status (Stankov et al., 2012). In addition, stigmatization, social isolation, or negative interpersonal relationships (Gray et al., 2008), may reinforce perceptions of low self-esteem and self-limitation in adolescents with overweight and obesity, at an important stage in their psychosocial development, thus amplifying their psychosocial vulnerability (Gmeiner and Warschburger, 2020).

Unfortunately, girls face greater barriers to physical activity than boys (Stankov et al., 2012).

The perception of reduced athletic capacity (Boyington et al., 2008), the negative perception of one’s own body, and the feeling of embarrassment about physical appearance are experienced as major barriers to physical activity in girls (Alm et al., 2008).

Since female students with obesity must be able to acquire skills and act like others in an environment adapted to their possibilities, we adjusted the content of basketball sessions to exercisers’ psychological and physiological profiles. However, while it is easy to find information on the causes of obesity, it is much more complicated to find adequate methods that can help teachers to act effectively with the student with obesity. In this regard, it should be considered that in inclusive teaching it is essential to encourage assertive communication and develop emotional skills, enhancing empathic capacity (Navarro-Mateu et al., 2019). Thus, empathy, which consists of emotional, cognitive empathy, and emotional disengagement, represents the ability to form and maintain relationships with others (Davis, 1983) and is closely related to children’s social competence (Sánchez-Pérez et al., 2014) and antisocial behavior (Eisenberg et al., 2010). Empathy also serves as an internal motivation for individuals to refrain from weight-based victimization (Shima et al., 2021), and is crucial in the inhibition of individual antisocial behavior (Baumeister and Lobbestael, 2011). It follows that students with high empathy are easily liked by others and often have good peer and teacher–student relationships (Boele et al., 2019; van den Bedem et al., 2019).

Some previous studies have shown that physical activity improves empathy in rodents (Yüksel et al., 2019), in people with multiple sclerosis (Sadeghi Bahmani et al., 2020), and in healthy young adults (Shima et al., 2021). However, no previous study has investigated the effect of an adapted PE session on the empathic ability of adolescents with obesity. Nevertheless, previous findings indicate that adolescent girls engaged in sports activities have a higher empathy than those who do not practice any kind of physical activity, and this is more evident following physical activity that requires greater cognitive involvement (Olander et al., 2013; Dishman et al., 2019). The data analyses of the present study indicated that adapted physical activity programs might have the power to favorably influence emotion regulation and empathy. By contrast, girls with obesity did not increase empathy during a classic basketball exercise (without adaptation), since no significant differences were recorded between before and after the intervention program. Although the school represents an important environment for student development and plays a key role in the growth process of the individual (World Health Organization, 1995), when the environment is not conducive to the situation of the students, particularly when considering obesity, a low level of empathy can be registered.

Since emotional contagion constitutes one of the foundations of empathy (Nakahashi and Ohtsuki, 2015), here we studied whether our intervention is useful for girls to increase emotional contagion. Emotional contagion has been defined as: “The tendency to automatically imitate and synchronize expressions, postures, and movements with those of another person and, consequently, to converge emotionally” (Hatfield et al., 1993). For example, during physical activity, subjects watching others automatically show increased activity in the same muscles that are being used by the target subjects (Arizmendi, 2011).

A previous study showed that playing about three minutes of basketball led to an increase, although not statistically significant, in emotional contagion. The lack of significance could be explained by the short duration of the activity. Indeed, our results show a significant increase in emotional contagion in girls who were engaged in a 7 week adapted basketball program. Generally speaking, emotional contagion and its effects on people’s individual and collective behaviors can influence work efficiency (Vijayalakshmi and Bhattacharyya, 2012). Consequentially, Nakahashi and Ohtsuki (2015) insisted that emotional contagion often works better in social learning strategy. Following the results of the present study, it appears necessary for the teacher to adapt their PE sessions for students with obesity, to let them benefit from the learning process when showing a good emotional state. These results are in line with those reported by Garel (2003) who considered that it is of great importance to think about exercise adaptation among students with obesity to facilitate their integration. In addition, Nazeri et al. (2019) showed that self-compassion and mindfulness training increased academic engagement and enjoyment in obese students during physical activity. Interestingly, this type of practice had a greater impact on the academic engagement increase among these students.

Regarding emotional splitting, i.e., a psychological mechanism that allows tolerating difficult emotions and facilitating their management (Carser, 1979), this is the first study to demonstrate that adapted physical activity can decrease emotional splitting in overweight girls. It is helpful to recall an earlier study showing that split scores in young women are directly proportional to the degree of obesity. These findings had significant implications for the treatment of obese patients due to possible influences on psychotherapy (Zmolikova et al., 2016). Although much evidence shows that PE provides skills that improve adaptability to social environments (Wan et al., 2021), little is known about its effects on students’ prosocial behaviors. In the case of children with autism it has been observed that different types of PE (for example aquatic exercises) have a positive effect on social cognition by improving their social interactions (Marzouki et al., 2022). PE is likely to improve social performance by modifying sensory perception and/or acting on brain regions involved in social behavior in both healthy and psychiatric individuals. In this regard, the activation of mirror neurons is the mechanism that would explain how exercise modifies social behaviors such as imitation, empathy, and emotional contagion (Xu et al., 2019).

The intervention showed here improved some dimensions of emotion regulation and empathy, so future studies may consider other important factors and data that are not included in this study and represent some limitations concerning a more detailed knowledge of the psychosocial inclusion process. In addition, the quality of the data does not allow for a deeper understanding of the reason for the reported positive responses (e.g., correlation with the improvement of body image disturbances, through a possible decrease in BMI through physical education). These points deserve future exploration. Likewise, our findings should be interpreted in the context of important limitations such as, to begin with, the age groups in which psychological and psychosocial needs differ. Secondly, the sample size was limited due to the difficulty of recruiting a more adequate number of overweight people in study classes taught by the same PE teacher. Therefore, further investigations with larger groups, including males, and of different ages, are warranted to evaluate physiological responses during adapted PE.

## 5. Conclusion

Unfortunately, in many countries, the conventional physical education program, and the outdated teacher curriculum, do not consider the physical condition and psychosocial limitations (i.e., empathic capacity) of students with obesity. We have here highlighted how adapted PE sessions closely correlate with the development of empathic capacity as evidenced by increased emotional contagion and decreased splitting emotions in adolescent girls with overweight. This result can be used by all teachers, who already play an important role in adapted physical education sessions, to enable overweight students to show more empathy. It is therefore hoped to include students with obesity in adequate and adapted exercises to obtain better results. To summarize, this teaching-learning intervention based on a basketball adaptation could be an effective strategy to allow integration into PE sessions of students with overweight.

## Data availability statement

The raw data supporting the conclusions of this article will be made available by the authors, without undue reservation.

## Ethics statement

The study was conducted in accordance with the Declaration of Helsinki, and approved by the Institutional Review Board (or Ethics Committee) of Higher Institute of Sport and Physical Education of Sfax University Sfax, Tunisia. Written informed consent to participate in this study was provided by the participants’ legal guardian/next of kin.

## Author contributions

OuS, AM, SM, and MB: conceptualization. OuS, OkS, and NJ: methodology. OkS, VC, and OuS: formal analysis. OuS and MB: writing—original draft preparation. AM, VC, and SM: writing—review and editing. AM and NJ: project administration. AM: supervision. All authors contributed to the article and approved the submitted version.

## Conflict of interest

The authors declare that the research was conducted in the absence of any commercial or financial relationships that could be construed as a potential conflict of interest.

## Publisher’s note

All claims expressed in this article are solely those of the authors and do not necessarily represent those of their affiliated organizations, or those of the publisher, the editors and the reviewers. Any product that may be evaluated in this article, or claim that may be made by its manufacturer, is not guaranteed or endorsed by the publisher.
